# SMCHD1 accumulates at DNA damage sites and facilitates the repair of DNA double-strand breaks

**DOI:** 10.1242/jcs.140020

**Published:** 2014-05-01

**Authors:** Heather Coker, Neil Brockdorff

**Affiliations:** Department of Biochemistry, University of Oxford, South Parks Road, Oxford OX1 3QU, UK

**Keywords:** Structural maintenance of chromosomes, SMC, SMCHD1, DNA repair, Double-strand breaks

## Abstract

SMCHD1 is a structural maintenance of chromosomes (SMC) family protein involved in epigenetic gene silencing and chromosome organisation on the female inactive X chromosome and at a limited number of autosomal loci. Here, we demonstrate that SMCHD1 also has a role in DNA repair of double-strand breaks; SMCHD1 is recruited to sites of laser micro-irradiated damage along with other DNA repair factors, including Ku80 (also known as XRCC5 in mammals) and RAD51. Cells deficient in SMCHD1 show evidence of decreased efficiency of repair and cell viability after DNA damage. We suggest that SMCHD1 responds to DNA double-strand breaks in a manner that is likely to involve its ability to alter chromatin states to facilitate DNA repair.

## INTRODUCTION

SMCHD1 is a non-canonical member of the structural maintenance of chromosomes (SMC) protein family that plays fundamental roles in higher-order chromosome organisation. SMCHD1 shares limited similarity with other SMC proteins, differing in ATPase domain structure and its inability to form a stable multimeric complex. SMCHD1 uniquely affects epigenetic gene silencing, chromosome organisation and CpG DNA methylation on the female inactive X chromosome and at a limited number of autosomal loci (Blewitt et al., 2005; Nozawa et al., 2013; Gendrel et al., 2013; Arne et al., 2013).

There is evidence for the participation of cohesin and the SMC5–SMC6 complex in homologous recombination (HR)-mediated DNA double-strand break (DSB) repair (Potts et al., 2006; Stephan et al., 2011; Watrin and Peters, 2009). Similarly, the *Arabidopsis thaliana* SMCHD1 homologue has been implicated in HR (Böhmdorfer et al., 2011). Here, we show that mammalian SMCHD1 is recruited to laser micro-irradiated DNA damage with dynamics suggestive of a role facilitating both non-homologous end joining (NHEJ) and HR-mediated DNA repair. SMCHD1 colocalises with Ku80 (also known as XRCC5 in mammals) at early stages of DNA repair, but can also persist at damage sites for 14 h or longer and colocalise with Rad51. In the absence or depletion of SMCHD1, significantly more DNA double-strand breaks are unresolved, and we observe diminished 53BP1 recruitment and decreased cell viability after DNA damage. These results demonstrate an important role for SMCHD1 in the mammalian DNA damage response. We suggest that SMCHD1 responds specifically to DNA damage in a manner that is likely to involve its ability to alter chromatin states and facilitate repair.

## RESULTS AND DISCUSSION

### Initial recruitment of SMCHD1 to DNA damage exhibits fast dynamics, but SMCHD1 enrichment persists in some cells

**Fig. 1.**
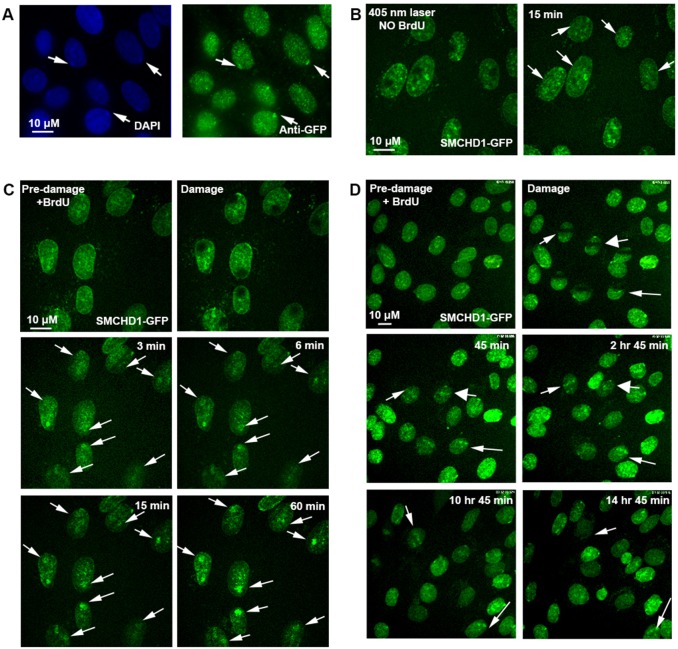
**SMCHD1 recruitment to sites of DNA damage after laser micro-irradiation.** In SD10 female fibroblast cells (A), DAPI staining identifies the compact Xi. Immunofluorescence identifies correct localisation of SMCHD1–GFP to the Xi (arrows). (B) Imaging of SMCHD1–GFP 15 min after laser microirradiation at the indicated sites in five cells, without pre-sensitisation with BrdU. (C) Live-cell imaging capturing recruitment of SMCHD1–GFP to laser micro-irradiated damage. Sites of damage in eight cells are indicated with arrows. Timepoints at 3, 6, 15 and 60 min after damage. (D) Live-cell imaging of SMCHD1–GFP recruitment to lines of laser micro-irradiated damage. Timepoints at 45 min, 2 h 45 min, 10 h 45 min and 14 h 45 min are shown. The broad short arrow indicates a cell in which SMCHD1 enrichment is lost by 2 h 45 min. The short thin arrow indicates a cell losing SMCHD1 enrichment before entering mitosis at 14 h 45 min, whereas the long thin arrow indicates a cell in which SMCHD1 persists.

As a first step towards investigating the role of SMCHD1 in DNA repair, we assessed the accumulation of GFP-tagged human SMCHD1 in BrdU pre-sensitised mammalian female SD10 fibroblast cells in response to DSB-inducing laser micro-irradiation (Kong et al., 2009; Mortusewicz and Leonhardt, 2007). We verified correct nuclear localisation of SMCHD1–GFP by visualising enrichment on the inactive X chromosome (Xi) (Gendrel et al., 2012) (Fig. 1A). Using live imaging, we observed BrdU-dependent SMCHD1 enrichment at sites of damage that were first evident after 3 min and persisted for at least 1 h (Fig. 1B,C). Recruitment of SMCHD1 was highly reproducible, with 82% of cells from six biological replicas demonstrating enrichment within 15 min. Rapid recruitment of SMCHD1 is consistent with a role in the early stages of repair, indeed, longer timecourses additionally demonstrated that SMCHD1 enrichment in some cells was lost by 2 h 45 min (indicative of NHEJ resolution of DSBs; Fig. 1D, short broad arrow). However, in other cells SMCHD1 enrichment persisted up to or longer than 14 h (Fig. 1D, short thin and long thin arrows, respectively). These observations indicate that whereas SMCHD1 is initially recruited to DNA damage with dynamics consistent with NHEJ, its persistence also suggests a role in later stages of repair.

### SMCHD1 colocalises with Ku80, but at later stages after DNA damage can colocalise with RAD51

**Fig. 2.**
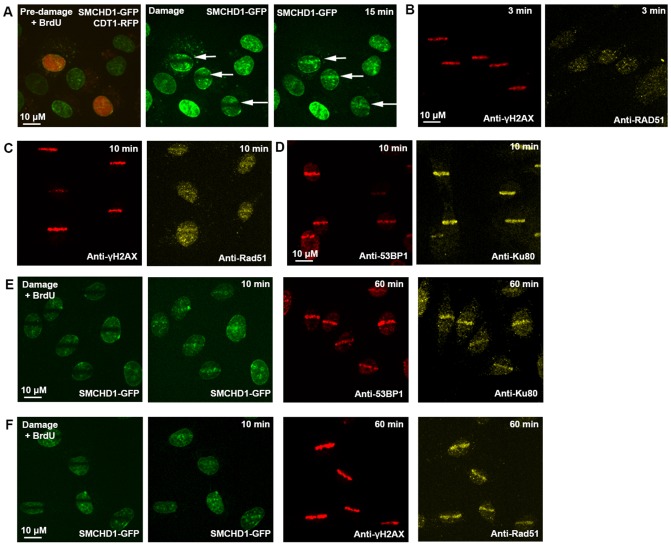
**Enriched SMCHD1 colocalises with early- and late-phase DNA repair factors at DNA damage sites.** In female SD10 fibroblast cells (A) transient CDT–RFP transfection enabled live detection of G1/S cells (red). Laser micro-irradiation of lines of damage in the two G1/S cells and a neighbouring cell with no CDT-RFP signal, indicated by arrows, led to recruitment of SMCHD1 to damage in all cases. (B) 3 min after laser micro-irradiation, immunofluorescence demonstrates γH2AX but no RAD51 recruitment. C) 10 min after laser micro-irradiation, immunofluorescence demonstrates γH2AX and faint RAD51 recruitment. (D) 10 min after laser micro-irradiation, immunofluorescence demonstrates 53BP1 and intense Ku80 recruitment. (E) Colocalisation of live SMCHD1–GFP (10 min) with 53BP1 and Ku80 by immunofluorescence, 60 min after damage. (F) Colocalisation of live SMCHD1–GFP (10 min) with γH2AX and RAD51 by immunofluorescence, 60 min after damage.

In order to understand the role of SMCHD1 in relation to different stages of DNA repair we utilised immunofluorescence to firstly show that SMCHD1 was recruited to damage sites in G1/S (HR deficient) cells (Fig. 2A) and was not confined to a role in HR. Then, using a combination of live SMCHD1–GFP imaging and fixed immunofluorescence analysis of DNA repair factors, we analysed recruitment of the HR factor RAD51 to sites of damage. RAD51 recruitment is not evident 3 min after damage (Fig. 2B), when γH2AX (Fig. 2B) and SMCHD1 (Fig. 1C) were already enriched. RAD51 showed a faint recruitment to damage after 10 min, in contrast with strong enrichment of the NHEJ component Ku80 at 10 min (with γH2AX and 53BP1) (Fig. 2C,D). At 1 h after damage [when SMCHD1 enrichment was still observed (Fig. 1C)], Ku80 enrichment appeared to be diminished, whereas RAD51 was prominent (Fig. 2E,F). SMCHD1 colocalised with the same DNA damage sites as Ku80, γH2AX and 53BP1 (Fig. 2E,F) but also with RAD51, which is recruited later (Fig. 2F). These data support a role for SMCHD1 in facilitating DNA repair mediated by both NHEJ and HR.

### *Smchd1-*null cells show aberrant γH2AX, consistent with inefficient DSB repair

**Fig. 3.**
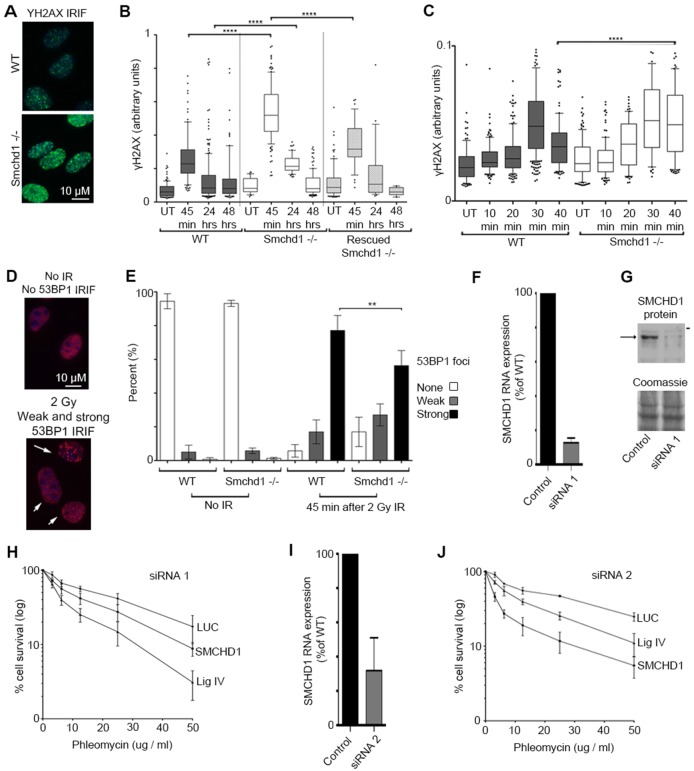
**The absence or depletion of SMCHD1 results in inefficient DNA repair and reduced cell viability after DNA damage.** (A) Immunofluorescence shows γH2AX foci 45 min after 2 Gy ionising radiation in WT and *Smchd1*^−/−^ MEFs. (B) Box and whisker plot illustrating quantitative analysis of γH2AX in WT, *Smchd1*^−/−^, or rescued *Smchd1*^−/−^ cells, 45 min, 24 h, 48 h after 10 Gy ionising radiation, or without damage, after immunofluorescence. Whiskers indicate the 10th–90th percentile, the box, the 25th–75th percentile, and the line the median. Outliers are indicated. *n* = 15–215 cells per timepoint. Statistical significance calculated using two-tailed unpaired Student's *t*-test. ****P<0.0001. (C) Box and whisker plot as in B, illustrating γH2AX in WT, or *Smchd1*^−/−^ cells harvested 10, 20, 30 or 40 min after 2 Gy ionising radiation, or without damage. Statistical significance calculated using an unpaired two-tailed Student's *t*-test. ****P<0.0001. (D) Categorisation of 53BP1 foci in WT MEFs as absent, e.g. in the absence of ionising radiation, as weak foci (small arrows) or strong foci (long arrow) after damage with 2 Gy ionising radiation and immunofluorescence. (E) Analysis of 53BP1 foci intensity in WT and *Smchd1*^−/−^ MEFs in the absence of damage and 45 min after 2 Gy ionising radiation. Statistical significance calculated using a unpaired two-tailed Student's *t*-test. **P<0.0022. Five repeat experiments for no ionising radiation, six repeat experiments with ionising radiation, 200 cells scored per condition per experiment. Results are mean±s.d. White bars represent percentage of cells with no 53BP1 foci, grey bars cells with weak foci and black bars those with strong foci. (F) qRT-PCR analysis of SMCHD1 RNA 72 h after siRNA 1 knockdown, normalised to HPRT and shown as a proportion of SMCHD1 in mock-siRNA-treated cells. Results are mean±s.d. of three experiments. (G) Western blot of SMCHD1 protein expression, 72 h after siRNA 1 knockdown with mock or SMCHD1 siRNA. SMCHD1 is indicated with the arrow, a 250 kDa marker with a horizontal dash. Equal loading was ensured by comparison of signal intensity of a Coomassie-stained portion of the same gel. (H) Cell survival assay demonstrating survival of SMCHD1 siRNA 1 (squares) or DNA ligase IV (Lig IV) (triangles) depleted U2OS cells relative to the siRNA control [luciferase siRNA (LUC) circles] after exposure to increasing phleomycin concentrations for 1 h. Results are mean±s.d. Each experiment contained four biological replicas, with experiments conducted in quadruplicate. (I) qRT-PCR analysis of SMCHD1 RNA 72 h after siRNA 2 knockdown, normalised to HPRT and shown as a proportion of SMCHD1 in mock siRNA treated cells. Results are mean±s.d. of three experiments. (J) Cell survival assay demonstrating survival of U2OS cells transfected with SMCHD1 siRNA 2 (squares), DNA ligase IV (Lig IV) (triangles) or siRNA control (luciferase siRNA (LUC) circles) after exposure to increasing phleomycin concentrations for 1 h. Results are mean±s.d. Each experiment contains four biological replicas, with experiments conducted in quadruplicate.

We then analysed the γH2AX response to Cs-137 ionising radiation in *Smchd1*-null (−/−) and wild-type (WT) mouse embryonic fibroblasts (MEFs) (Löbrich et al., 2010). Initial qualitative assessment 45 min after damage indicated that there was a stronger γH2AX signal in *Smchd1*^−/−^ compared to WT MEFs (Fig. 3A). We then subjected asynchronous MEFs to 10 Gy ionising radiation, before analysing γH2AX foci by immunofluorescence. Quantitative analysis of γH2AX, 45 min, 24 and 48 h after damage demonstrated significantly more γH2AX in *Smchd1*^−/−^ compared to WT MEFs at 45 min and 24 h (two-tailed unpaired Student's *t*-test, *P*<0.0001 for both), although DSBs in *Smchd1*^−/−^ cells were resolved by 48 h (Fig. 3B). This delay in repair could be rescued by complementation with WT SMCHD1 (Fig. 3B) (γH2AX signal relative to *Smchd1*^−/−^ cells at 45 min *P*<0.0001, using a two-tailed unpaired Student's *t*-test).

As the majority of repair in an asynchronous population is mediated by NHEJ, we then analysed γH2AX in samples at 10, 20, 30 and 40 min post-damage using a reduced ionising radiation dose of 2 Gy (Fig. 3C). In WT MEFs, γH2AX fluorescence intensity increased for the first 30 min, in agreement with the continued expansion of γH2AX domains, but at 40 min early stage repair was evident, because DSBs marked by γH2AX had been resolved (Fig. 3C). In contrast, *Smchd1*^−/−^ MEFs showed a distinctly different profile, with a significant lag in resolution of DSBs observed at 40 min (*P*<0.0001, two-tailed unpaired Student's *t*-test).

### Formation of 53BP1 foci is diminished in *Smchd1*^−/−^ MEFs

Recruitment of 53BP1 promotes NHEJ by antagonising HR-initiating factors (Dimitrova et al., 2008; Bunting et al., 2010), but has also been proposed to facilitate HR in G2 cells (Kakarougkas et al., 2013). We reasoned that *Smchd1*^−/−^ MEFs, might therefore also exhibit an altered profile of 53BP1 recruitment to sites of damage. We scored 53BP1 recruitment, with cells categorised as having strong, weak or absent foci 45 min after 2 Gy ionising radiation (Fig. 3D). In the absence of ionising radiation, no 53BP1 foci were observed in almost 95% of WT and *Smchd1*^−/−^ MEFs (Fig. 3E). At 45 min after ionising radiation, 77% of WT MEFs responded with strong foci, 17% with weak or indistinct foci and 6% failed to generate any foci (Fig. 3E). In contrast, in *Smchd1*^−/−^ MEFs, there was a significant reduction to 56% of cells exhibiting strong foci, 27% of *Smchd1^−/−^* cells exhibiting weak foci, and 17% of cells with no recruitment of 53BP1 (two-tailed unpaired Student's *t*-test, *P*<0.0022 when comparing strong foci in WT versus *Smchd1*^−/−^ cells) (Fig. 3E). This result is consistent with our previous observations suggesting that cells lacking SMCHD1 have a diminished response to DNA damage.

### SMCHD1 deficiency reduces cell survival in response to DNA damage

In order to investigate the effect of SMCHD1 deficiency on cell viability, SMCHD1 was depleted in U2OS cells, using two different siRNAs. siRNA 1 reduced SMCHD1 RNA to 13% of its endogenous level (Fig. 3F) and protein to almost undetectable levels (Fig. 3G). SMCHD1 cells treated with siRNA 1 demonstrated significantly reduced viability (Fig. 3H), particularly at higher doses of phleomycin (Student's two-tailed paired *t*-test comparing SMCHD1 siRNA and mock siRNA treated cell survival, *P* = 0.003 at 12.5 µg/ml, 0.032 at 25 µg/ml and 0.047 at 50 µg/ml of phleomycin). SMCHD1 siRNA 2 [RNA reduced to 32% of endogenous (Fig. 3I)] also significantly reduced viability (Fig. 3J) (Student's two-tailed paired *t*-test comparing survival between cells treated with SMCHD1 siRNA and mock siRNA, *P* = 0.024 at 3.125 µg/ml and 0.002 or less at concentrations of 6.25–50 µg/ml of phleomycin).

### Conclusions

Although the canonical SMC complexes have a role in HR (Potts et al., 2006; Stephan et al., 2011; Watrin and Peters, 2009), here we demonstrate a role for mammalian SMCHD1 in facilitating both NHEJ and HR mediated DNA repair. Depletion of SMCHD1 results in delayed DSB repair and reduced cell viability. The alteration in early repair dynamics, as indicated by γH2AX persistence in *Smchd1^−/−^* MEFs, the initial timing of recruitment of SMCHD1 and its colocalisation with Ku80 are all suggestive of a role facilitating NHEJ. As the majority of cells in an asynchronous population undergo repair by NHEJ, this explains the strong effect of SMCHD1 loss on early stage repair. However, the persistence of SMCHD1 enrichment in some cells, its colocalisation with RAD51 and the alteration of γH2AX repair dynamics even 24 h after DNA damage suggests that SMCHD1 also impacts on later phases of DNA repair, including HR. Collectively these findings point to a role for mammalian SMCHD1, potentially by modulating the organisation of chromatin, in facilitating the resolution of DNA damage.

## MATERIALS AND METHODS

### Cell culture and transfection

Cells were cultured in DMEM with 10% FCS, 50 µg/ml penicillin-streptomycin, 2 mM L-glutamine, 1× non-essential amino acids and 50 µM 2-mercaptoethanol in a 37°C incubator and under 5% CO_2_. Details of SD10 fibroblast and MEF line derivation available upon request.

### Immunofluorescence

Cells were grown to a sub-confluent monolayer on glass coverslips. If appropriate, cells were subject to damage using ionising radiation (Caesium-137 Graviton), and allowed to recover. Coverslips were washed with PBS, fixed with 4% paraformaldehyde, permeabilised with 0.5% Triton X-100 and stained as standard. For immunofluorescence including Ku80 or RAD51, pre-extraction was carried out with CSK+R buffer prior to fixation (Britton et al., 2013). Cells were imaged using a Zeiss AX10 microscope equipped with an AxioCam MRM CCD camera and Axiovision software. CellProfiler 2.0 image analysis software was used for automated analysis (www.cellprofiler.org). Mouse monoclonal anti-GFP (1∶1000, Roche), mouse monoclonal anti-γH2AX (1∶1000, Upstate), rabbit polyclonal anti-53BP1 (1∶1000, Novus Biologicals), mouse monoclonal anti-Ku80 (1∶200, Thermo Scientific), rabbit polyclonal anti-Rad51 (1∶200), Alexa-Fluor-488-conjugated goat anti-mouse IgG (H and L), and Alexa-Fluor-568-conjugated goat anti-rabbit IgG (H and L) (1∶400, Molecular Probes) antibodies were used.

### Laser micro-irradiation

Cells were seeded in μ-dishes (Ibidi), pre-sensitised with 10 µg/ml BrdU for 24 h. For cell cycle analysis, 20 µl Premo Fucci Cell Cycle Sensor CDT-RFP mix (Life Technologies) was added to the cells in 1 ml medium 48 h prior to imaging. Cells were imaged in a humid 37°C Tokai chamber under 5% CO_2_. Cells were damaged using 100% 405-nm laser power, 50 iterations, and a damage focus of either 3-µm diameter, or a line of 1×100 pixels. Images were captured using 15% 488 laser power with 17 *z*-slices of 0.5-µm step size using a PerkinElmer spinning disk confocal microscope, ×60/1.4 NA oil immersion objective and a Hamamatsu C9100-13 EMCCD camera (either 512×512 pixels, or for the 15-h timecourse, 1024×1024 pixels). Volocity 6.1.1 software was used for acquisition and analysis, in conjunction with Image JA to produce projected images from composite *z* sections. For colocalisation of SMCHD1–GFP with repair factors by immunofluorescence, damage to cells in a single field of view was followed by a single snapshot confirming recruitment at 10 min and then immunofluorescence as detailed after 3 or 60 min, followed by further snapshots using the PerkinElmer spinning disk confocal microscope as detailed.

### Knockdown of human SMCHD1

125 pmoles of stealth siRNA HSS146332 (siRNA 1) or HSS146334 (siRNA 2) (Life Technologies), and, as a control, either the Low GC Duplex (Life Technologies) or siLUC was added to cells according to the manufacturer's instructions and incubated for 72 h. The following primers were used for standard qRT-PCR using SYBR green (BioRad), with HPRT1 for normalisation: smcHD1F, 5′-TCCGGATATGAGGAAGAAAAAG-3′; smcHD1R, 5′-TGTCGTCTCAACCTTTGGTG-3′, HPRTF; 5′-GACCAGTCAACAGGGGACAT-3′; HPRTR, 5′-CCTGACCAAGGAAAGCAAAG-3′. Standard western blot analysis of SMCHD1 expression used RIPA-generated whole-cell extract and anti-SMCHD1 (Bethyl, 1∶2000) followed by donkey anti-rabbit IgG HRP antibody (GE Healthcare, 1∶20,000) with Coomassie staining of part of the gel to visualise loading.

### Cell survival assay

96 h after the first of a double siRNA transfection over consecutive days, cells were plated into six-well plates at 500 cells per well in quadruplicate technical repeats. Cells were treated the next day for 1 h with phleomycin, washed once with warm PBS and refreshed with DMEM. After 10 days, colonies were stained with 0.5% Crystal Violet in 20% (v/v) ethanol and counted. Results were normalised to plating efficiency. Biological repeats were performed in quadruplicate.
